# Assessing the microbiota of recycled bedding sand on a Wisconsin dairy farm

**DOI:** 10.1186/s40104-021-00635-6

**Published:** 2021-11-11

**Authors:** Hannah E. Pilch, Andrew J. Steinberger, Donald C. Sockett, Nicole Aulik, Garret Suen, Charles J. Czuprynski

**Affiliations:** 1 Department of Pathobiological Sciences, School of Veterinary Medicine, University of Wisconsin-Madison, Madison, 53706 USA; 2 Department of Bacteriology, University of Wisconsin-Madison, Madison, 53706 USA; 3 Microbiology Doctoral Training Program, University of Wisconsin-Madison, Madison, 53706 USA; 4 Wisconsin Veterinary Diagnostic Laboratory, School of Veterinary Medicine, University of Wisconsin-Madison, Madison, 53706 USA

**Keywords:** Bovine, Dairy farm, Microbiota, Recycled bedding sand, 16S rRNA sequencing

## Abstract

**Background:**

Sand is often considered the preferred bedding material for dairy cows as it is thought to have lower bacterial counts than organic bedding materials and cows bedded on sand experience fewer cases of lameness and disease. Sand can also be efficiently recycled and reused, making it cost-effective. However, some studies have suggested that the residual organic material present in recycled sand can serve as a reservoir for commensal and pathogenic bacteria, although no studies have yet characterized the total bacterial community composition. Here we sought to characterize the bacterial community composition of a Wisconsin dairy farm bedding sand recycling system and its dynamics across several stages of the recycling process during both summer and winter using 16S rRNA gene amplicon sequencing.

**Results:**

Bacterial community compositions of the sand recycling system differed by both seasons and stage. Summer samples had higher richness and distinct community compositions, relative to winter samples. In both summer and winter samples, the diversity of recycled sand decreased with time drying in the recycling room. Compositionally, summer sand 14 d post-recycling was enriched in operational taxonomic units (OTUs) belonging to the genera *Acinetobacter* and *Pseudomonas,* relative to freshly washed sand and sand from cow pens. In contrast, no OTUs were found to be enriched in winter sand. The sand recycling system contained an overall core microbiota of 141 OTUs representing 68.45% ± 10.33% SD of the total bacterial relative abundance at each sampled stage. The 4 most abundant genera in this core microbiota included *Acinetobacter*, *Psychrobacter*, *Corynebacterium*, and *Pseudomonas*. *Acinetobacter* was present in greater abundance in summer samples, whereas *Psychrobacter* and *Corynebacterium* had higher relative abundances in winter samples. *Pseudomonas* had consistent relative abundances across both seasons.

**Conclusions:**

These findings highlight the potential of recycled bedding sand as a bacterial reservoir that warrants further study.

**Supplementary Information:**

The online version contains supplementary material available at 10.1186/s40104-021-00635-6.

## Background

Providing suitable housing and bedding is critical for maintaining quality of life for dairy cattle. Cattle that are comfortable and well cared produce more milk and are more profitable [1–3]. One method to increase cow comfort is to use appropriate bedding material. There are several bedding options available, including rubber mats, straw, wood shavings, dried manure solids, newspaper clippings, and sand. Many consider sand as the ideal bedding type because it improves cow comfort and resting time, and studies comparing sand to other bedding types show decreases in lameness and incidences of hock lesions [4–7]. Moreover, studies have also shown that total bacterial counts in sand bedding are lower than in other organic bedding types [8–11]. Given that organic bedding is often implicated in the spread of bacterial pathogens responsible for diseases such as mastitis [12], the use of sand bedding may help in mitigating such diseases.

In addition to the health and comfort benefits of sand bedding, it is also cost effective as sand can be recycled and reused. The process involves mechanically removing used sand from free stalls and then pushing the sand using recycled water (grey water) to a central drain which leads to a separate sand recycling room. In the recycling room, sand is separated from organic material, cleaned with a disinfectant, and left to dry in the recycling room. This method typically leads to a sand recovery rate of > 90% and several studies have shown recycled sand to have no significant difference in bacterial load, relative to clean sand [8, 13, 14]. However, these results contrast with a more recent survey of 169 U.S. dairy herds, which found unused recycled bedding sand to have increased total bacterial counts when compared to unused fresh sand [11]. Additionally, recycling bedding sand does not remove all organic material and some bacteria (e.g., coliforms, *Klebsiella pneumoniae*) are known to utilize the available and residual organic nutrients to proliferate [13]. As a result, it has been suggested that recycled bedding sand could serve as a potential reservoir for bacterial pathogens [11, 15].

To date, there is limited information regarding the microbiota of recycled bedding sand. Most prior studies have only utilized culture-based surveys to quantify bacterial colonies or counts of pathogens commonly implicated in mastitis [9, 11, 13, 14]. As a result, these studies may lack the ability to identify less common or unculturable environmental pathogens. Moreover, no prior work has been done to examine the broader bacterial communities of a recycled bedding sand system. Here, we hypothesized that the microbiota of bedding sand is dynamic across both season (summer vs. winter) and stage of the recycling process, and that a core microbiota exists and persists across all recycling stages and seasons. To test this, we characterized the microbiota of recycled bedding sand collected in both summer and winter on a dairy farm in South-central Wisconsin, USA, across different stages of the recycling process using 16S rRNA gene amplicon sequencing. We report the bacterial taxa conserved throughout the recycling process and determined whether the diversity of these bacterial communities is affected by season and recycling stage.

## Methods

### Recycled sand collection

Recycled bedding sand was collected at various points in the recycling process from a dairy farm (approximately 1,500 Jersey cows and heifers) in South-central Wisconsin, USA. In brief, the recycling process involves removal of the top layer of sand from free stalls; thereafter, it is mechanically pushed into the aisle and flushed into a central drain using recycled water (“grey water”). Grey water is water stored in the sand recycling room used exclusively to move sand through the recycling system and is itself constantly reused across many recycling productions. The sand and grey water then travel through a drain into a central reception pit located in a dedicated recycling room in a separate building on the farm. The sand is allowed to settle in the reception pit and filled with grey water. Organic material is siphoned off and sent for composting. Sand is removed from the bottom of the pit using a commercial rotating auger. As the sand moves upward through the auger, it is doused with a disinfectant containing hypochlorous acid prior to being deposited into a large “wet recycled sand” pile. The wet recycled sand piles are approximately 2 m tall and 5–6 m wide. Once deposited, these piles remain stationary and are allowed to air-dry indoors in the recycling room for 11–14 d; they are then redistributed to re-bed free stalls on an as need basis. A full diagram of this process is presented in Supplementary Fig. S1. At the time of this study, the farm did not utilize commercial sand dryers, nor did they implement any process to hasten the sand drying process.

Sand samples were collected using sterile wooden tongue depressors (McKesson Medical-Surgical Inc., Richmond VA) and placed into sterile 50-mL conical tubes (Falcon, Fisher Bioscience, Waltham MA). At each location of the recycling process, five separate 5 mL sand samples were collected and used to create a single composite 25 mL sand sample. This was done in triplicate at all sand sampling locations except for cow pens where a total of 5 composite samples were collected from randomly chosen locations in the free stall cow pens. Sand was collected as available once in May 2018 (“summer”) and again in January 2019 (“winter”). Summer sand samples were collected from cow pens, from a pile of freshly washed wet recycled sand, a sand pile 4 d post-recycling (“4-d”) (from the surface of the pile and 7 in subsurface), a sand pile 14 d post-recycling (“14-d”) (from the surface and 7 in subsurface), and a pile of new sand that had not yet been used or recycled. In addition, approximately 40 mL of grey water was collected directly into a 50-mL conical tube, in triplicate, from the central holding tank of the sand recycling system. Winter samples were collected from a pile of freshly washed wet recycled sand, a sand pile 2 d post-recycling (“2-d”) (from the surface and 7 in subsurface), and a sand pile 7 d post-recycling (“7-d”) (from the surface and 7 in subsurface). Approximately 40 mL of grey water was also collected directly into 50-mL conical tubes, in triplicate, from the reception pit used to separate sand from organic material. In the winter we were unable to collect sand samples at the same timepoints as summer (4-d and 14-d post recycling) as the sand is processed for recycling on an as-needed basis. Sand was not collected from free stalls during the winter due to logistical constraints. After each collection, samples were transported on ice to our laboratory and stored at − 80 °C.

### DNA extraction, amplification, and sequencing

All samples were thawed to room temperature and processed individually. For sand samples, 3 mL of sand was vortexed in 4 mL DNA extraction buffer and 1 mL of the resultant liquid was used for DNA extraction [16]. For grey water, DNA was extracted directly from 1 mL of sample. Total genomic DNA was extracted for each sample individually using a mechanical disruption and hot/cold phenol and phenol:chloroform extraction as previously described [16], with the modification of 25:24:1 phenol:chloroform:isoamyl alcohol in place of phenol:chloroform. DNA samples were resuspended in Tris Buffer and quantified with a Qubit 2.0 fluorometer (Thermo Fisher Scientific, Waltham, MA). The V4 hypervariable region of the bacterial 16S rRNA gene was amplified by polymerase chain reaction (PCR) using barcoded, 1-step sequencing primers as previously described [17]. PCR reactions were performed using 25 ng template DNA, 0.5 μL forward and reverse primers at 10 mmol/L, 12.5 μL KAPA 2xHiFi Master Mix (Roche, Rotkreuz, Switzerland), and nuclease free water (IDT, Coralville, Iowa, USA) to a total volume of 25 μL. Grey water samples were processed identically to sand samples. Water was used in place of template DNA for PCR negative controls, which were processed identically to sand samples.

PCR reaction conditions were as follows: 95 °C for 3 min, 25 cycles of 95 °C for 30 s, 55 °C for 30 s, and 72 °C for 30 s, followed by a final extension at 72 °C for 5 min. PCR products were quantified on a 1% (wt/vol) low-melt agarose gel, desired PCR products (~ 380 bp) were excised and recovered via gel extraction using a 96-well Zymoclean Gel DNA Recovery Kit (Zymo Research, Irvine, CA), and quantified using a Qubit High-Sensitivity assay kit (Thermo Fisher Scientific) and a Synergy 2 Multi-Mode plate reader (BioTek, Winooski, VT). DNA samples were equimolarly pooled to 4 nmol/L and sequenced on an Illumina Miseq (Illumina, Inc., San Diego, CA) using a 500-cycle MiSeq v2 sequencing kit with custom sequencing primers [17].

### Sequence cleanup and classification

Paired-end reads were demultiplexed on the Illumina MiSeq and processed using mothur v.1.44.1 [18], as previously described [17]. Briefly, paired-end reads were assembled into contiguous sequences and those having ambiguous base calls, homopolymers longer than 8 bp, and lengths less than 200 bp or greater than 500 bp were removed. Sequences were aligned to the V4 region of the SILVA 16S rRNA gene reference database (v138) [19] and those not aligned were removed. Chimeric sequences were detected using Uchime [20] and removed. Taxonomic classification was performed using the SILVA taxonomic database (v138) with an 80% minimum confidence cut-off. Sequences classified as unknown, Archaea, Eukaryota, chloroplast, or mitochondria were removed, as were singleton sequences. Operational taxonomic units (OTUs) were clustered at 97% sequence similarity using the OptiClust method [21] and were taxonomically classified against the SILVA database with 80% minimum confidence. Representative sequences were determined using “get.oturep” with method = distance and were further taxonomically identified using blastn against the National Center for Biotechnology Information’s (NCBI) nucleotide database [22]. Samples with Good’s coverages, estimates of sample diversity captured by sequencing data, < 0.95 were removed [23]. Resultant samples were normalized to 16,000 sequences per sample. Alpha diversity metrics, Chao’s Richness and Shannon’s Diversity Index, and post-normalized Good’s coverage were calculated in mothur from the normalized OTU table. The sequencing dataset supporting the conclusions of this article is available in the NCBI’s Sequence Read Archive under BioProject number PRJNA724660 [https://www.ncbi.nlm.nih.gov/bioproject/PRJNA724660].

### Sequence analysis in R

Statistical analyses were performed in R (v4.1.0) [24]. Bacterial communities were assessed between seasons (summer or winter), between sampling locations for each season individually (summer: cow pen, grey water, wet recycled sand, 4-d sand, and 14-d sand; winter: grey water, wet recycled sand, 2-d sand, 7-d sand), and between depths (surface or 7-in subsurface). When comparing depths, data were subset to only locations having both surface and 7-in samples (summer: 4-d and 14-d; winter: 2-d and 7-d) and were compared separately for summer and winter samples. For subsequent analyses, surface and subsurface samples for each location were grouped together as there was no significant difference in bacterial communities of these locations. When testing seasonal effects on alpha and beta diversity, summer cow pen samples were removed as they had no analogue in the winter sample set and the remaining locations were seasonally grouped as there were no direct analogs between summer and winter locations. Alpha diversity was assessed using Shannon’s Diversity Index, a measure of richness and evenness, and Chao’s Richness estimate. Normality was assessed using the Shapiro-Wilk test. Significance was determined using ANOVA or the Kruskal-Wallis (KW) test, depending on normality. Post-hoc pairwise comparisons were similarly performed using Tukey’s HSD or the Wilcoxon Rank Sum test in the case of significance *(P <* 0.05).

Beta-diversity was evaluated using Bray-Curtis dissimilarity, calculated using the R package vegan v2.5–7 [25], and both unweighted and weighted UniFrac metrics, calculated using a rooted neighbor-joining tree of OTUs in the R packages phangorn::import_mothur_dist (v2.7.1) [26], ape::bionj (v5.5) [27], and phyloseq::UniFrac (v1.36.0) [28, 29]. Beta-diversity was visualized in nonmetric multi-dimensional scaling (nMDS) plots with square root transformed data using vegan and ggplot2 v.3.3.5 [30]. Beta-dispersion was tested (vegan::betadisper) and, finding no violation of this assumption in our analyses, permutational multivariate ANOVA (vegan::adonis2) was used to assess significance. Pairwise comparisons were assessed using vegan::adonis2 and *P*-values were FDR-corrected for multiple comparisons. OTUs driving community differences between sampling groups were identified by the similarity percentages (SIMPER) function (vegan::simper) for seasonal and location analyses. OTUs explaining > 1% of the difference between groups were subjected to Kruskal-Wallis tests, with FDR-correction applied to confirm differential abundance with *P* < 0.05.

The core bacterial community was assessed by first identifying the OTUs present in all individual samples from each sampling location. These OTUs were then compared across all summer and winter sampling locations. Those found in all locations were considered the “core” microbiota. Heat trees describing the core microbiome were generated from a neighbor-joining tree of OTUs using the heat_tree function from the R package metacoder [31]. Differential abundance of core taxa between summer and winter was assessed using the Wilcoxon rank sum test with FDR correction. Log2 ratio of median relative abundance counts for taxa with adjusted *P* > 0.05 were adjusted to zero for plotting.

### Bacterial plating and counts

Presence of viable bacteria was assessed for one sample collected from several sampled locations. Samples were thawed and 5 mL of each were placed into sterile 50-mL conical tubes. Immediately after, 45 mL of PBS was added before being vortexed. After mixing, 0.1 mL was removed and serially diluted in PBS. Each dilution was then plated on trypticase soy agar with 5% sheep’s blood (Becton Dickson, Franklin Lakes, NJ) and incubated at 37 °C for 24 h before counting colony forming units (CFUs) to determine total aerobic bacterial counts.

## Results

### Sampling and sequencing summary

A total of 1,833,757 raw sequences were generated from 26 summer samples (947,496 sequences), 18 winter samples (884,245 sequences), and 4 PCR negative controls (2,016 sequences). After cleanup, 1,390,389 sequences remained, including 736,707 from summer, 653,099 from winter, and 583 from the negative controls, resulting in 7,159 unique OTUs representing 34 phyla, 419 families, and 920 genera. The 2 never-before-used summer sand samples (SHP2, SHP16) and the 4 PCR negative controls were removed during normalization of sample sequence counts to 16,000 sequences per sample, with each having < 220 sequences per sample. This resulted in 24 summer and 18 winter samples that were used in all subsequent analysis. Pre-normalized Good’s coverages of the remaining samples ranged from 0.969 to 0.992 and post-normalized Good’s coverages ranged from 0.936 to 0.984. See Supplementary Table S1, Additional File 2 for additional details. The resulting summer samples averaged 16,072 ± 159 SD sequences per sample from 5,423 unique OTUs. Winter samples averaged 15,969 ± 197 SD sequences per sample from 4,488 unique OTUs.

### Bacterial diversity and sand depth

Bacterial community diversity was first compared between samples collected at the surface and 7-in below the surface of drying recycled sand piles. Analyses were performed separately for summer and winter samples. No differences in alpha diversity were found across sampling depths for either summer (Chao: *P* = 0.15, KW, Shannon: *P* = 0.52, KW) or winter samples (Chao: *P* = 0.15, KW, Shannon: *P* = 0.09, ANOVA). Alpha diversity metrics are visualized in Supplementary Fig. S2, Additional File 1. Bray-Curtis Dissimilarity also did not differ by depth in either summer (*P* = 0.54, ANOVA) or winter samples (*P* = 0.14, ANOVA), as visualized in Supplementary Fig. S3, Additional File 1. Similar results were obtained using UniFrac, both weighted (summer: *P* = 0.39, winter: *P* = 0.14, ANOVA) and unweighted (summer: *P* = 0.65, winter: *P* = 0.17, ANOVA). As a result, surface and 7-in samples were combined for subsequent analyses.

### Bacterial diversity across seasons

Comparison of bacterial community compositions between summer and winter samples reveal seasonal differences. Taxonomically, the 6 phyla with the highest relative abundances in all summer samples were the Proteobacteria (37% ± 15% SD), Bacteroidetes (30% ± 9%), Firmicutes (22% ± 13%), Actinobacteria (6% ± 4%), Spirochaetota (1% ± 2%), and Campilobacterota (1% ± 1%). The 5 genera with the largest relative abundances include the *Acinetobacter* (17% ± 12%), *Psychrobacter* (3% ± 2%), *Pseudomonas* (3% ± 2%), *Proteiniphilum* (3% ± 2%), and *Flavobacterium* (2% ± 1%). Similarly, winter samples were dominated by bacteria in the phyla Firmicutes (35% ± 10%), Proteobacteria (25% ± 8%), Bacteroidetes (24% ± 5%), Actinobacteria (12% ± 5%), Campilobacterota (2% ± 1%), and Spirochaetota (1% ± 1%). The 5 genera with the highest relative abundances in winter include *Psychrobacter* (7% ± 4%), *Corynebacterium* (6% ± 3%), *Erysipelothrix* (5% ± 1%), an unclassified Lactobacillales (5% ± 2%), and *Facklamia* (4% ± 2%). See Supplementary Figs. S4 and S5, Additional File 1, for more detail on bacterial community compositions.

Seasonal alpha and beta diversity analyses were performed without summer cow pen samples as there was no corresponding winter samples collected. For alpha diversity, which describes the diversity of individual samples, we found Chao’s Richness, an estimate of the number of unique species in a sample, to be greater in summer samples relative to winter samples *(P =* 0.025, KW). Shannon’s Diversity, which considers both the richness and evenness of a sample, did not differ by season *(P =* 0.239, ANOVA), suggesting summer samples contain additional rarer OTUs than winter samples (Supplementary Fig. S6, Additional File 1). Beta diversity, which describes the differences in community composition between samples, was determined using the Bray-Curtis Dissimilarity, a metric that considers both richness and evenness, and revealed the bacterial community composition of summer and winter samples to be distinct (*P* < 0.001, ANOVA) (Supplementary Fig. S7, Additional File 1). Similar results were also obtained using weighted and unweighted UniFrac distances (both *P* < 0.001, ANOVA), metrics that consider OTUs taxonomic relatedness in addition to richness (unweighted) and both richness and evenness (weighted). A total of 13 OTUs were predicted by SIMPER to drive the differences between summer and winter bacterial communities, of which 11 were differentially abundant by season. Of those 11, only 3 had higher mean relative abundances in summer than in winter: OTU1 and OTU2, which both classified to the genus *Acinetobacter*, and OTU15 classified to the genus *Proteiniphilum*. The remaining 8 OTUs were found in higher mean relative abundances in the winter samples, including OTU5, which classified as belonging to the genus *Corynebacterium*. See Supplementary Table S2, Additional File 2, for additional detail. Summer and winter samples were analyzed separately for subsequent analyses.

### Bacterial diversity of summer sampling locations

**Fig. 1:**
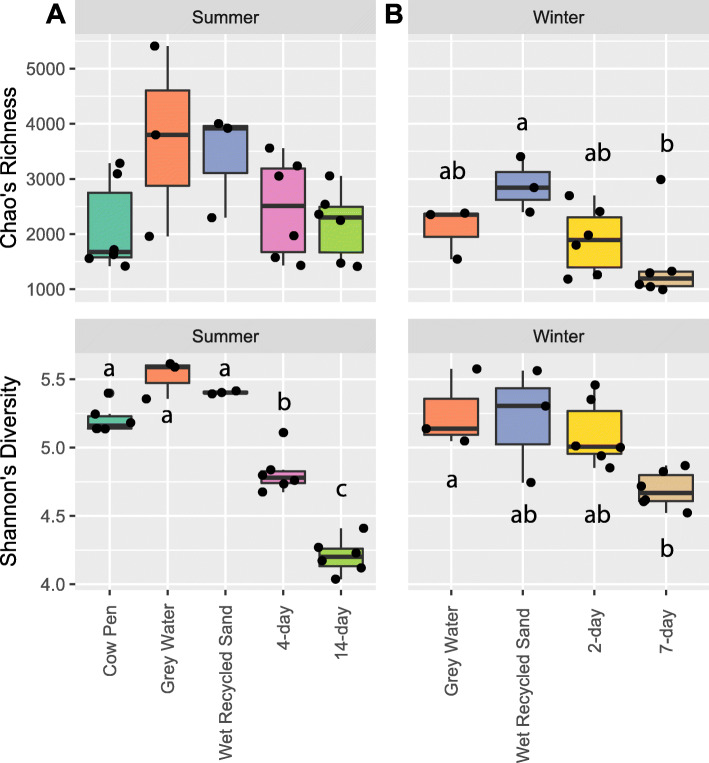
Alpha diversity of summer and winter sampling locations. Boxplots of Chao’s Richness and Shannon’s Diversity Index values of samples from each of the summer (**A**) and winter (**B**) sampling locations. Differing letters indicate a significant difference between locations within each season (*P* < 0.05) using the Wilcoxon Rank Sum test for summer (**A**) and Tukey’s HSD for winter (**B**) locations. Pairwise comparisons of Chao’s Richness were not performed for summer locations since no overall differences were found (*P* > 0.05). Surface and 7-in samples are not differentiated as no differences were found between them across locations (*P* > 0.05)

**Fig. 2:**
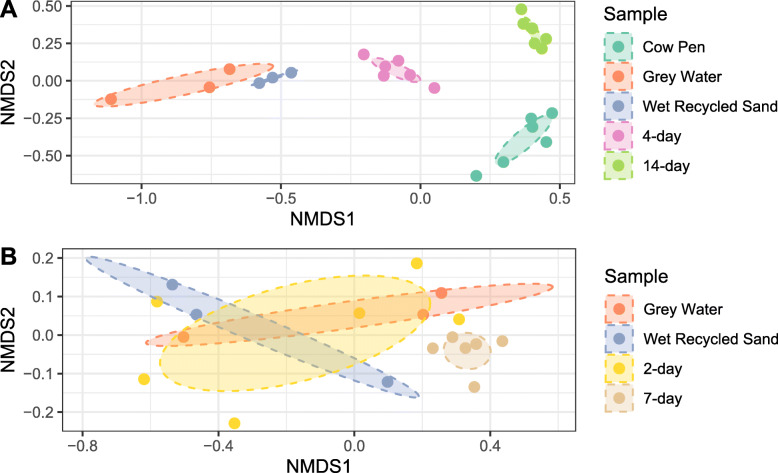
Differences of bacterial community compositions across summer and winter sampling locations. Non-metric multidimensional scaling (nMDS) plot of the Bray-Curtis dissimilarity for microbial communities of recycled sand samples colleted in (**A**) summer and (**B**) winter. Each dot represents the bacterial community of a single sample and are colored by location. Ellipses represent standard error around centriod of each location and are colored by location. Surface and 7-in samples are not differentiated as no differences were found between them across locations (*P* > 0.05). Stress: A = 0.07, B = 0.052

The microbial diversity was compared across the 5 summer sampling locations: cow pens, grey water, wet recycled sand, 4-d sand, 14-d sand. Chao’s Richness did not differ across locations (*P* = 0.185, KW) (Fig. 1A). Shannon’s Diversity differed across sampling locations (*P* = 2.9e-3, KW) with the cow pen, grey water, and wet recycled sand samples being the most diverse. 4-d sand had the next highest diversity, being significantly lower diversity than the first 3 locations, and 14-d sand being the least diverse (Fig. 1A). The Bray-Curtis dissimilarity also differed between locations (*P* = 1e-4, ANOVA), with all 5 sampling locations being distinct except for the grey water and wet recycled sand samples (*P* = 0.1, ANOVA) (Fig. 2A and Supplementary Table S3, Additional File 2). Similar results were obtained using weighted and unweighted UniFrac and can be found in Supplementary Table S3, Additional File 2. A total of 34 OTUs were found to be differentially abundant between locations. Specifically, 6 OTUs were in lower abundance in 14-d sand compared to cow pen samples, including those in the genera *Moheibacter*, *Ornithobacterium*, and *Aequorivita*. We found 3 OTUs increased in abundance in the 14-d sand samples, relative to cow pen sand, including OTU1 and OTU2, which both classified to the genus *Acinetobacter,* and OTU3, which classified to the genus *Pseudomonas.* For additional detail, see Supplementary Table S4, Additional File 2.

### Bacterial diversity of winter sampling locations

Bacterial community diversity was compared across the 4 winter sampling locations: grey water, wet recycled sand, 2-d sand, and 7-d sand. Community diversity differed across winter sampling locations, but to a lesser extent than summer samples. Both Chao’s Richness (*P* = 0.048, ANOVA) and Shannon’s Diversity (*P* = 0.015, ANOVA) differed slightly across the 4 winter locations (grey water, wet recycled sand, 2-day sand, and 7-day sand). Pairwise comparisons of individual locations revealed that Chao’s Richness of wet recycled sand was greater than 7-d sand (*P* = 0.03, Tukey HSD), Shannon’s Diversity of grey water was greater than 7-d sand (*P* = 0.026, Tukey HSD), and Shannon’s Diversity of wet recycled sand and 2-d sand trended towards being greater than 7-d sand (*P* = 0.055 and *P* = 0.059 resp., Tukey HSD) (Fig. 1B). Bray-Curtis dissimilarity and weighted UniFrac distances between winter locations trended towards a difference (*P* = 0.059 and *P* = 0.051 resp., ANOVA) while unweighted UniFrac distances did differ across locations (*P* = 0.005, ANOVA). Pairwise comparisons found 7-d sand to differ from both grey water and 2-day sand samples for all metrics, and from wet recycled sand when using unweighted UniFrac (Fig. 2B and Supplementary Table S3, Additional File 2). However, no OTUs predicted by SIMPER were differentially abundant between locations after FDR correction (Supplementary Table S5, Additional File 2].

### A Core microbiota

**Fig. 3:**
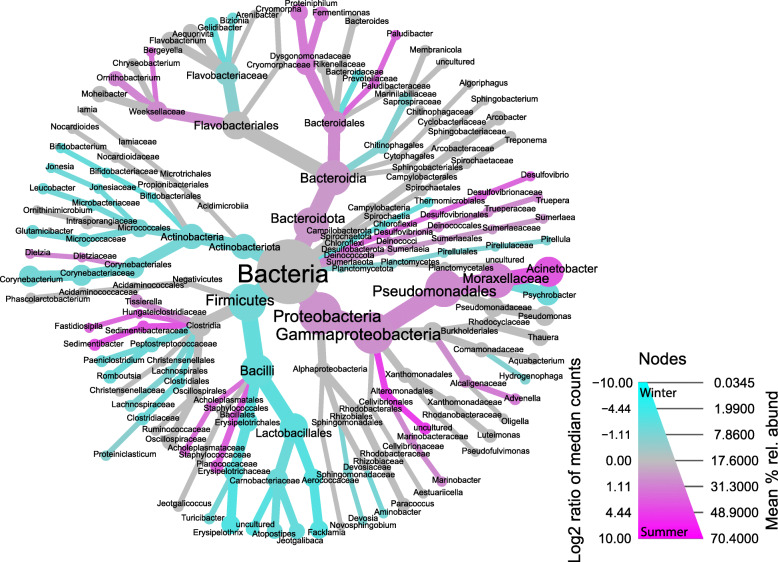
The core microbiota of a bedding sand recycling system. A heat-tree of the core microbiota of a bedding sand recycling system. Node and line widths increase with the mean relative abundance of the core taxonomic groups across all samples. Colors indicate the log2 ratio of the median counts of taxanomic groups between summer and winter samples. Increasing magenta indicates increased abundance in summer samples, cyan for increased abundance in winter samples, and grey represents no seasonal difference. Log2 ratio of median counts were set to zero for taxnomic groups having non-significant differential abundance by season (*P* < 0.05), per FDR-corrected pairwise Wilcoxon rank sum tests of median abundance counts

The core microbiota was determined, which consisted of 141 OTUs belonging to 12 phyla and 97 genera and represents 68.45% ± 10.33% SD of the relative bacterial abundance of each sampling location. Across sampling locations, the relative abundance of the core microbiota ranged from 44.8% to 78.1% of summer locations and 67.9% to 80.2% of winter locations. The most abundant genera of the core microbiota include *Acinetobacter* (10% ± 12%), *Psychrobacter* (5% ± 4%), *Corynebacterium* (4% ± 3%), *Pseudomonas* (3% ± 3%), and an unclassified Lactobacillales (3% ± 2%). To better resolve the taxonomic identity of the core OTUs initially identified as “unclassified Lactobacillales” (OTU4 and OTU123), we conducted a BLASTN analysis of their consensus sequences against the NCBI’s non-redundant (nr) nucleotide database. The OTUs had a 98.42% and 99.21% sequence identity match, respectively, to several species in the genus *Enterococcus*, suggesting that these unclassified Lactobacillales likely belong to this genus. We also found these abundant core genera to be differentially abundant across seasons (Fig. 3). Of the most abundant genera, *Acinetobacter* was more abundant in summer than in winter samples, whereas *Psychrobacter, Corynebacterium*, and the unclassified Lactobacillales (*Enterococcus*) were more abundant in winter samples. Core OTUs classified to the genus *Pseudomonas* were not differentially abundant between seasons.

### Viable bacterial counts

Viable aerobic bacterial counts were assessed for all summer sampling locations and two winter locations. Recovery from summer sand sampling locations ranged from 1.06 × 10^7^ to 9.18 × 10^8^ CFU/g of sand and 6.39 × 10^6^ CFU/mL from summer grey water. Winter grey water resulted in 2.98 × 10^7^ CFU/mL and winter 7-d surface sand contained 5.40 × 10^7^ CFU/g. The remaining winter sand samples were not available for this analysis. See Supplementary Table S6, Additional File 2 for additional detail.

## Discussion

In this study, we sought to characterize the bacterial community composition of a bedding sand recycling system on a Wisconsin dairy farm with respect to both location and season. Our compositional analysis revealed the 6 most abundant phyla for summer and winter samples to be identical, suggesting broad seasonal commonality. The most abundant phyla included several often found in the environment and in the bovine gastrointestinal tract including the Bacteroidetes, Firmicutes, Spirochaetota, Campilobacterota (formerly class Epsilonproteobacteria), and Proteobacteria, as well as the primarily soil-associated phylum Actinobacteria (Supplemental Figs. S3 and S4, Additional File 1) [32–40]. Both seasons were dominated by the Bacteroidetes, Firmicutes, and Proteobacteria, with the Proteobacteria being the most abundant phylum in summer samples and Firmicutes being the most abundant in winter samples. These results are largely consistent with those of a recent study focused on the microbiota of recycled bedding sand and its association with hock lesions and digital dermatitis in dairy cattle [41], although in their study, Actinobacteria was the most abundant phylum. This discrepancy may be due to their bedding system, which consisted of sand on top of compact soils, rather than concrete, thereby increasing the prevalence of soil Actinobacteria in their sand samples. This discrepancy may also result from several additional factors including variability in sand recycling system, season of collection, farming practices, or the surrounding environment.

Of the top 5 genera for each season, the relative abundances of *Acinetobacter*, *Pseudomonas*, and *Corynebacterium* are of particular note as these genera contain species that are known pathogens of dairy cattle and prior work has suggested that bacteria in the environment or bedding can be a leading cause of diseases on dairy farms [8, 42]. In our analysis, *Acinetobacter* was the most abundant genus across the sand recycling system. *Acinetobacter* are aerobic, rod-shaped Gram-negative bacteria, and notable for their ability to acquire resistance to multiple classes of antibiotics and survive for long periods of time on dry surfaces [43–45]. Though widely distributed in nature and not considered major mastitis pathogens in the US, studies of Chinese and Korean dairy herds have found *Acinetobacter* spp. to be increasingly isolated from mastitic milk samples [46–49]. Hoque et al. also found *Acinetobacter* to be the predominantly abundant genus in milk from Indian dairy cattle with clinical mastitis using shotgun metagenomics [50].

Similarly, *Pseudomonas* and *Corynebacterium* also contain species that are environmentally distributed and are known causative agents for bovine mastitis, specifically *P. aeruginosa* and *C. bovis* [51, 52]. Given the prevalence of *Pseudomonas* in our analysis, we note that the farm sampled in this study experienced cases of mastitis believed to be caused by *Pseudomonas* during our sampling period (D. Sockett, WI Veterinary Diagnostic Laboratory, personal communication, 2018). Although the methods used in our study are unable to conclusively differentiate taxa to the species-level, our findings suggest a potential route of transmission for *Pseudomonas* as a mastitis pathogen, and future work using culture-based approaches is warranted.

We next sought to understand how season impacted microbiota composition. Our data reveal that summer samples have increased richness, relative to winter samples, but we found no seasonal difference in Shannon’s Diversity metric, which considers both richness and abundance. This indicates that summer samples are primarily enriched in low abundance taxa and that the overall relative abundances of community members do not significantly shift between seasons (Supplementary Fig. S6, Additional File 1). Although the relative abundances did not strongly differ, the compositions of these communities differed between seasons (Supplementary Fig. S7, Additional File 1), which agrees with a previous study reporting that bacterial populations of bedding materials vary seasonally, likely due to changes in temperature and humidity [9, 53]. We note that the average temperature and humidity in May in southern Wisconsin is 20 °C and 66% humidity while in January the average temperature is − 3 °C and 75% humidity [54]. Given these findings, we posit that the microbial variation we observed is due to changes in overall temperature and humidity, and that this extends to all stages of the sand recycling process.

Our analyses further revealed enrichment of *Acinetobacter* OTUs in summer samples and the enrichment of several OTUs, including one *Corynebacterium* OTU, in winter samples (Supplemental Table S2, Additional File 2). These findings are also consistent with a previous report indicating that culturable mastitis pathogen counts increase in summer months [9]. Although less work has been done to understand the seasonal dynamics of *Acinetobacter* and *Corynebacterium* in dairy bedding, the incidences of human clinical infections of *Acinetobacter baumannii* are known to greatly increase in summer months, as are the relative abundances of freshwater *Acinetobacter* spp. [55, 56]. Similarly, a survey of milk isolates collected by the Wisconsin Veterinary Diagnostic Lab from 1,994 to 2,001 found the incidence of *Corynebacterium bovis* isolations to differ seasonally, though with higher incidences in summer months than in winter months [57].

Comparisons of bacterial community compositions between stages of the sand recycling system revealed high heterogeneity in summer samples but less heterogeneity in winter. For the summer samples, bacterial communities of the cow pen, grey water, and freshly recycled sand all had equally high diversity which then declined in the 4-d sand and further declined in the 14-d sand (Fig. 1A). Community compositions were similarly stratified with 4-d and 14-d communities being distinct from grey water and freshly recycled sand; however, communities of cow pen samples were also distinct from all other locations (Fig. 2A). These results suggest that the bacterial communities in cow pen sand are significantly modified by the sand recycling process. Identification of the bacterial taxa driving the differences between cow pen sand and 14-d sand (sand that is ready for re-bedding) revealed only 6 OTUs to have reduced abundance in 14-d sand relative to cow pen sand. Surprisingly, 3 OTUs were enriched in 14-d sand relative to sand from cow pens, 2 of which were classified as *Acinetobacter* and the third as *Pseudomonas*. Importantly, both *Acinetobacter* and *Pseudomonas* increased in relative abundance with time in the recycling room, with a 2–4-fold increase in *Acinetobacter* abundance and a 7-fold increase in *Pseudomonas* abundance (Supplementary Fig. S4, Additional File 1). These results may reflect that both organisms are known biofilm producers, which may allow them to resist removal from sand granules during washing, providing a competitive advantage [51, 58, 59]. Additionally, *Acinetobacter* spp. such as *A. baumannii* are well characterized as having high survivability on dry surfaces for long periods [44, 45]. However, the moisture content of the sand was not directly measured so additional studies are required to confirm the impact of moisture in this system. Additionally, with our current sampling design, we cannot discern whether these genera are surviving washing and then re-colonizing the sand or are being introduced from the surrounding farm environment as the sand sits in piles the sand recycling room.

Our study also found the bacterial communities in winter samples to be less heterogeneous across sampling locations, with only 7-d sand having decreased richness and diversity and a distinct community composition from several other sampled locations (Figs. 1B and Fig. 2B). No OTUs were found to drive the differences in diversity between 7-d sand compositions and 2-d sand, grey water, or wet recycled sand compositions, after correcting for multiple testing (Supplementary Table S5, Additional File 2). This is likely due to a combination of low sample size and reduced heterogeneity in the winter samples. The impact of drying on winter sand, especially relative to summer samples, is difficult to determine in our study as dryness was not measured and we performed 1-day convenience sampling and were subject to sand availability on that day. Similarly, cow pen samples were not readily available for winter sampling, preventing comparison of the community composition of 7-d sand with cow pen sand. We additionally found no difference in bacterial diversity or composition of sand collected at the top of sand piles and sand collected at lower depths for any of the samples despite the sand at the top being dry and the sand within the pile being visibly wet (Supplementary Figs. S2 and S3, Additional File 1).

A final goal of our study was to determine if a core microbiota was present across the sand recycling system. Members of the core microbiota we identified accounted for a surprisingly large portion of the bacterial community relative abundance for all locations (mean 68.45% ± 10.33% SD), which suggests a large portion of the recycled sand microbiota may be stable and consistent across seasons. The top 4 genera of the core microbiota included *Acinetobacter*, *Corynebacterium*, *Psychrobacter*, and *Pseudomonas*, with *Acinetobacter* enriched in summer samples, *Corynebacterium* and *Psychrobacter* enriched in winter, and *Pseudomonas* not differentially abundant by season, matching our earlier seasonal comparison of the total bacterial community (Fig. 3).

In addition, the most abundant genera identified in this study, *Acinetobacter*, *Corynebacterium, Psychrobacter, Pseudomonas,* and *Enterococcus*, are also common organisms associated with bulk tank raw milk [48, 60–63]. Although this study did not assess the microbial quality of the milk, the environment in which cattle are housed has been implicated as a source of milk contamination [64, 65]. While sand is viewed an ideal choice for bedding because it carries a decreased bacterial load in comparison to organic bedding materials, recycling sand may not necessarily eliminate the risk of bulk tank milk contamination.

We suggest that the presence and prevalence of several bacterial genera known to cause mastitis and bulk tank contamination warrant further investigation of the recycled sand microbiota, as it is possible that sand recycling does not eliminate sand bedding as a potential reservoir for these pathogens. As such, future work should seek to increase the number of farms to determine the variability of recycled sand communities across different farms and sand recycling systems. We note that the results of our study are constrained to the analysis of bacterial DNA relative abundances. While the enumeration of bacterial microflora in sand was found to be approximately 10^7^–10^8^ colony forming units per gram of sand, the incorporation of qPCR combined with a larger sample set from several farms would provide quantitative descriptions of the effects of sand recycling on total and specific bacterial abundances. Culturing and/or qPCR could thereby confirm if mastitis or enteric pathogens can be viably passaged through the sand recycling system across seasons. We also note that is study is unable to definitively identify the mechanisms driving seasonal and locational differences in bacterial community composition, thereby requiring more controlled future studies to discern the roles of dryness, climate, and environment in shaping this system. Finally, several of the most abundant genera we identified are characterized by their ability to accrue multi-drug resistance [51, 59, 66]. Focused work on the antibiotic resistance gene (ARG) carriage of both pathogenic and commensal bacteria could reveal the role of recycled bedding sand systems in ARG accumulation and dissemination.

## Conclusions

In conclusion, we found that the bacterial community composition of a WI dairy farm bedding sand recycling system is both dynamic across seasons and stages of the sand recycling process, with a large “core” microbiota accounting for most of the bacterial community reads at each sampling location. The identification of an abundant core microbiota, and the presence and enrichment of several genera with known mastitis pathogen species suggests that bedding sand recycling systems may serve as bacterial reservoirs that warrant further investigation.

## Supplementary Information

**Additional file 1: Supplementary Fig. S1** Additional File 1.docx; Diagram of the sand recycling process and its stages. **Supplementary Fig. S2** Additional File 1.docx; Violin plots of Chao’s Richness (A) and Shannon’s Diversity (B) of samples collected from the surface (top) of the drying recycled sand piles or 7-in (7in) below the surface. **Supplementary Fig. S3** Additional File 1.docx; Non-metric multidimensional scaling (nMDS) plot of the Bray-Curtis dissimilarity of microbial communities from the surface (top) and 7-in subserface (7in) samples of drying recycled sand piles. **Supplementary Fig. S4** Additional File 1.docx; Stacked bar plots of the relative abundances of the 6 most abundant phyla (A) and 10 most abundant genera (B) in grey water and sand at all locations in the recycling process during summer sampling. **Supplementary Fig. S5** Additional File 1.docx; Stacked bar plots of the relative abundances of the 6 most abundant phyla (A) and 10 most abundant genera (B) in grey water and sand at all locations of the recycling process during winter sampling. **Supplementary Fig. S6** Additional File 1.docx; Violin plots of Chao’s Richness (A) and Shannon’s Diversity (B) of samples collected in summer and winter. **Supplementary Fig. S7** Additional File 1.docx; Non-metric multidimensional scaling (nMDS) plot of the Bray-Curtis dissimilarity for microbial communities of recycled sand samples collected during summer and winter sampling.

**Additional file 2: Supplementary Table S1** Additional File 2.xlsx; A table of samples, their coverage, read counts, alpha diversity metrics, and metadata. **Supplementary Table S2** Additional File 2.xlsx; List of OTUs predicted by SIMPER to drive differences found between summer and winter samples. **Supplementary Table S3** Additional File 2.xlsx; *P*-values of pair-wise comparisons of Bray-Curtis Dissimilarity and UniFrac distances between summer and winter locations. **Supplementary Table S4** Additional File 2.xlsx; List of OTUs predicted by SIMPER to drive differences found between summer sampling locations. **Supplementary Table S5** Additional File 2.xlsx; List of OTUs predicted by SIMPER to drive differences found between winter sampling locations. **Supplementary Table S6** Additional File 2.xlsx; A table of aerobic viable plate counts for several summer and winter sampling locations.

## Data Availability

The datasets generated and analyzed during the current study are available in the NCBI Sequence Read Archive repository under BioProject number PRJNA724660 [https://www.ncbi.nlm.nih.gov/bioproject/PRJNA724660].
