# Supplementary material for: Assessing the microbiota of recycled bedding sand on a Wisconsin dairy farm
Source: J Anim Sci Biotechnol. 2021 Nov 11;12:114. doi: 10.1186/s40104-021-00635-6 (PMC8582206; doi:10.1186/s40104-021-00635-6)
Supplement: Supplementary file 1 — Additional file 1: Supplementary Fig. S1 Additional File 1.docx; Diagram of the sand recycling process and its stages. Supplementary Fig. S2 Additional File 1.docx; Violin plots of Chao’s Richness (A) and Shannon’s Diversity (B) of samples collected from the surface (top) of the drying recycled sand piles or 7-in (7in) below the surface. Supplementary Fig. S3 Additional File 1.docx; Non-metric multidimensional scaling (nMDS) plot of the Bray-Curtis dissimilarity of microbial communities from the surface (top) and 7-in subserface (7in) samples of drying recycled sand piles. Supplementary Fig. S4 Additional File 1.docx; Stacked bar plots of the relative abundances of the 6 most abundant phyla (A) and 10 most abundant genera (B) in grey water and sand at all locations in the recycling process during summer sampling. Supplementary Fig. S5 Additional File 1.docx; Stacked bar plots of the relative abundances of the 6 most abundant phyla (A) and 10 most abundant genera (B) in grey water and sand at all locations of the recycling process during winter sampling. Supplementary Fig. S6 Additional File 1.docx; Violin plots of Chao’s Richness (A) and Shannon’s Diversity (B) of samples collected in summer and winter. Supplementary Fig. S7 Additional File 1.docx; Non-metric multidimensional scaling (nMDS) plot of the Bray-Curtis dissimilarity for microbial communities of recycled sand samples collected during summer and winter sampling. [file 40104_2021_635_MOESM1_ESM.docx]

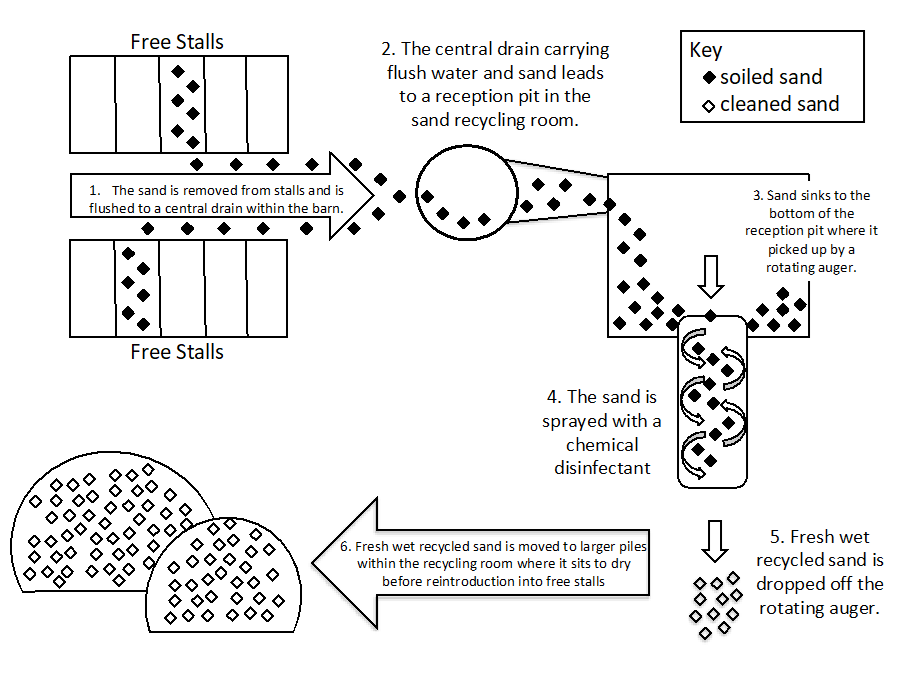
Supplementary Figure S1. Diagram of the sand recycling process and its stages.


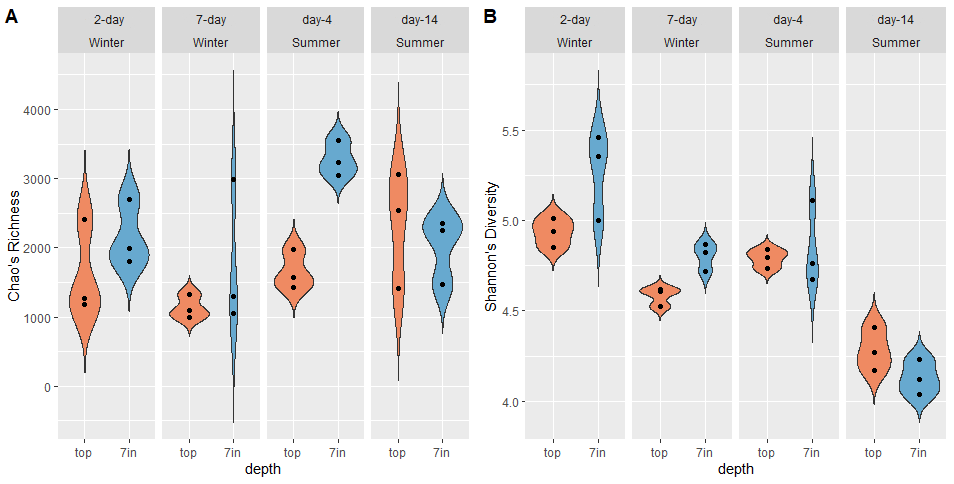


Supplementary Figure S2. Violin plots of Chao’s Richness (A) and Shannon’s Diversity (B) of samples collected from the surface (top) of the drying recycled sand piles or 7-in (7in) below the surface, colored by depth (top=orange, 7in=blue). * *P*<0.05. None were significant.


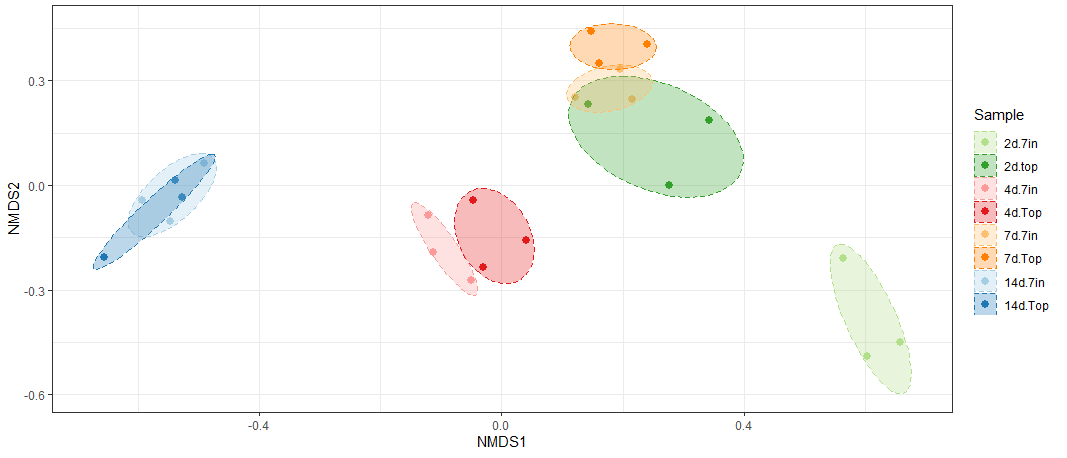


Supplementary Figure S3. Non-metric multidimensional scaling (nMDS) plot of the Bray-Curtis dissimilarity of microbial communities from the surface (top) and 7-in subsurface (7in) samples of drying recycled sand piles. Each dot represents bacterial community of a single sample and are colored by location and depth collected. Ellipses represent standard error around centriod of each location-depth combination and are colored by location-depth combination. Stress=0.078.


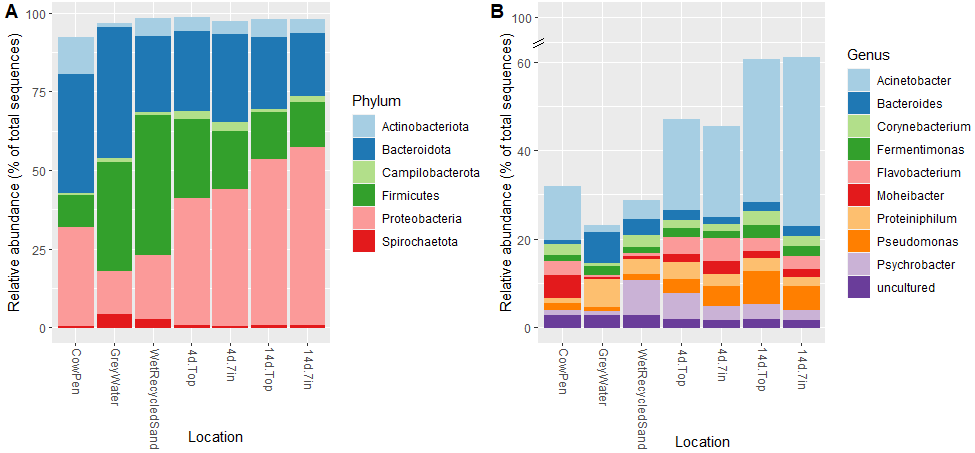


Supplementary Figure S4. Relative abundances of the 6 most abundant phyla (A) and 10 most abundant genera (B) in grey water and sand at all locations of the recycling process during summer sampling.


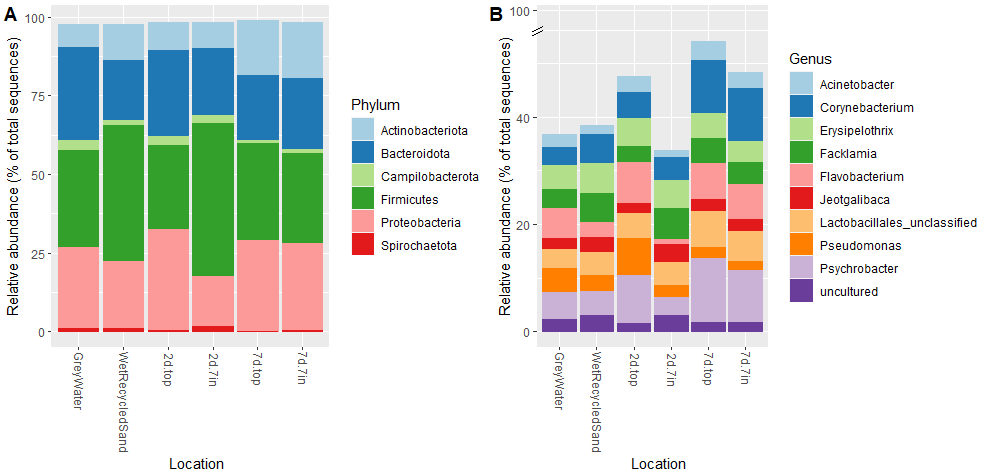
Supplementary Figure S5. Relative abundances of the 6 most abundant phyla (A) and 10 most abundant genera (B) in grey water and sand at all locations of the recycling process during winter sampling.


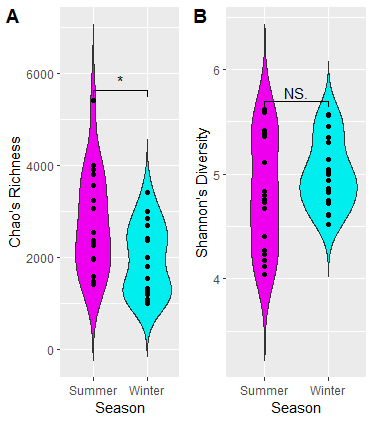


Supplementary Figure S6. Violin plots of Chao’s Richness (A) and Shannon’s Diversity (B) of samples collected in summer and winter, colored by season (summer=magenta, winter=cyan). Summer cow pen samples were not included. Surface and 7-in subsurface samples were not distinct (*P*>0.05) and are not distinguished here for readability. * *P*<0.05, NS. *P*>0.05.


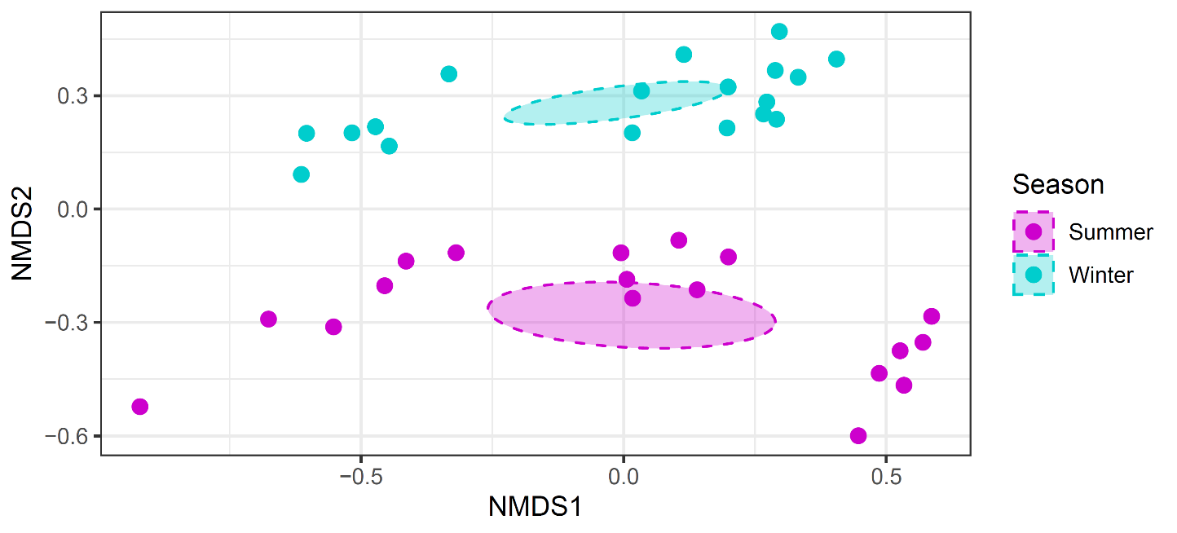


Supplementary Figure S7. Non-metric multidimensional scaling (nMDS) plot of the Bray-Curtis dissimilarity for microbial communities of recycled sand samples. Each dot represents the bacterial community of a single sample, ellipses represent standard error around centroid of each season, and both are colored by season. Summer cow pen samples were not included. Surface and 7-in subsurface samples were not distinct (*P*>0.05) and are not distinguished here for readability. Stress=0.084.
